# Characterization of MazF-Mediated Sequence-Specific RNA Cleavage in *Pseudomonas putida* Using Massive Parallel Sequencing

**DOI:** 10.1371/journal.pone.0149494

**Published:** 2016-02-17

**Authors:** Tatsuki Miyamoto, Yuka Kato, Yuji Sekiguchi, Satoshi Tsuneda, Naohiro Noda

**Affiliations:** 1 Department of Life Science and Medical Bioscience, Waseda University, 2–2 Wakamatsu-cho, Shinjuku-ku, Tokyo, 162–8480, Japan; 2 Biomedical Research Institute, National Institute of Advanced Industrial Science and Technology (AIST), 1-1-1 Higashi, Tsukuba, Ibaraki, 305–8566, Japan; University of Manchester, UNITED KINGDOM

## Abstract

Under environmental stress, microbes are known to alter their translation patterns using sequence-specific endoribonucleases that we call RNA interferases. However, there has been limited insight regarding which RNAs are specifically cleaved by these RNA interferases, hence their physiological functions remain unknown. In the current study, we developed a novel method to effectively identify cleavage specificities with massive parallel sequencing. This approach uses artificially designed RNAs composed of diverse sequences, which do not form extensive secondary structures, and it correctly identified the cleavage sequence of a well-characterized *Escherichia coli* RNA interferase, MazF, as ACA. In addition, we also determined that an uncharacterized MazF homologue isolated from *Pseudomonas putida* specifically recognizes the unique triplet, UAC. Using a real-time fluorescence resonance energy transfer assay, the UAC triplet was further proved to be essential for cleavage in *P*. *putida* MazF. These results highlight an effective method to determine cleavage specificity of RNA interferases.

## Introduction

Toxin-antitoxin (TA) systems are genetic modules composed of a stable toxin and a volatile antitoxin. They are widely distributed among archaeal and bacterial lineages and allow microbes to withstand environmental stresses [1–3]. When microbes face these environmental stresses, antitoxin, which prevents toxin activity, is rapidly degraded. Then, toxin molecules inhibit requisite cellular functions, causing microbial growth arrest and eventually cell death [4]. Although the molecular functions of toxins are diverse, a very large number of toxins across the archaeal and bacterial lineages are known to function as endoribonucleases [5,6]. In *Escherichia coli*, several types of toxin ribonucleases have been shown to catalyze intracellular RNA cleavage in a ribosome dependent (RelE, HigB, YafO, YafQ, and YoeB) [7–11] or independent (MazF, MqsR, ChpBK, HicA, and RnlA) [12–16] fashion.

MazF, which is one of the most investigated RNA interferases, is a part of a TA system along with its cognate antitoxin MazE [4]. As with other TA systems, the lethality of MazF is triggered by various stresses such as antibiotic addition, heat shock, and amino acid starvation [17,18]. In such situations, MazE is degraded by ClpAP protease [4], releasing the MazF toxin endoribonuclease. This liberated MazF subsequently cleaves intracellular RNA at ACA triplets [12] and permits cells to change their translation pattern [19,20]. To date, many MazF homologues were predicted in the genomes and plasmids of various prokaryotes [21], and some of them have been characterized. Intriguingly, their cleavage patterns differed between microbes in terms of recognition-site length (in most cases three, five, or seven bases) and RNA sequences [12,22–27]. These reports indicate that RNA interferases are diverse, even within one MazF family, and that these proteins play broad roles in degrading the bulk of intracellular RNA and/or silencing specific genes to cope with the environmental fluctuations [28,29].

The cleavage patterns produced by most RNA interferases are yet to be identified because of limitations from using classical methods. In most studies, primer extension analysis with MS2 bacteriophage RNA and chaperone protein CspA has been used [22,26,27]. Although it enables direct detection of RNA cleavage sites, the experimental method is cumbersome and impractical to adapt for high-throughput studies. Mass spectrometry was also developed as a rapid, sensitive, and accurate method for detection of cleavage sites [30], however, one major drawback of this approach is that mass resolution decreases as RNA oligonucleotide length increases [31]. Gel electrophoresis is one of the simplest methods to observe the cleaved products, but it is difficult to identify the sequence of the cleavage sites without prior information. To overcome these limitations, a novel method termed MORE (mapping by overexpression of an RNase in *E*. *coli*) RNA-seq was developed [32]. In this method, RNA interferase was ectopically overexpressed in *E*. *coli* and the 5′-end sequences of the extracted RNAs that correspond to the cleavage sequence were detected with SOLiD platform [32]. This method is useful to accurately identify the cleavage sequence of RNA interferases, particularly for cleavage sequences longer than five nucleotide bases.

In our study, we also determined the cleavage sequence of RNA interferases using RNA-seq. The RNAs used in this study were designed with a computer algorithm that generates diverse sequences [33], and thus, the RNAs can be studied for many potential cleavage sequences in a high-throughput manner with the Illumina MiSeq platform. When we tested our approach with *E*. *coli* MazF, ACA was identified as the cleavage sequence. Thus, to verify this approach in another microbe, an uncharacterized MazF homologue that is predicted in the *Pseudomonas putida* chromosome was also analyzed. The RNA-seq showed that UAC was the RNA cleavage sequence. This observation was further confirmed by a fluorometric assay [34], demonstrating the efficacy of this approach for identifying RNA interferase-specific cleavage sequences.

## Materials and Methods

### Bacterial Strains and Plasmids

The following *E*. *coli* strains were used in this study: DH5α (Nippon Gene, Tokyo, Japan) for the constructed plasmid propagation and BL21 (DE3) pLysS (Novagen, Madison, WI, USA) for recombinant protein expression. Two plasmids were purchased from Takara Bio Service, pMD19 encoding the *graA* gene and empty vector pET21c (Takara, Shiga, Japan).

### Oligonucleotides

PCR primers were purchased from Tsukuba Oligo Service (Ibaraki, Japan). All fluorescent-labeled oligonucleotides were purchased from Japan Bio Services (Saitama, Japan). Barcode RNA was also purchased from Japan Bio Services (Saitama, Japan). The sequence of PCR primers and a barcode RNA are listed in S1 Table.

### Plasmid Construction

PCR was carried out to amplify the antitoxin (*mazEpp*) and the toxin (*mazFpp*) genes with Taq HS DNA polymerase (Takara) using genomic DNA of *Pseudomonas putida* as a template. These PCR products were purified by a PCR purification kit (Qiagen, Venlo, Netherlands). pET21c and PCR products were digested with *Bam*HI and *Eco*RI (Toyobo, Osaka, Japan) and cleaned using phenol/chloroform extraction and ethanol precipitation. Digested *mazEpp* and *mazFpp* fragments were inserted into the corresponding pET21c multiple cloning sites with a DNA ligation kit (Takara). This generated the plasmids pET21c-*mazEpp* and pET21c-*mazFpp*, respectively. Each plasmid was then introduced into *Escherichia coli* DH5α and the colonies were grown overnight at 37°C on LB plate containing ampicillin (100 μg/ml). Plasmids were extracted using QIAprep Spin Miniprep Kit (Qiagen) and their sequences were determined using AB 3500 Genetic Analyzer (Applied Biosystems, Foster City, CA, USA) according to the manufacturer’s protocol.

### Protein Expression

The pET21c-*mazEpp* and pET21c-*mazFpp* plasmids were introduced into *E*. *coli* BL21 (DE3) pLysS via heatshock. The *E*. *coli* harboring pET21c-*mazEpp* or pET21c-*mazFpp* were pre-cultivated in LB medium containing 100 μg/ml ampicillin at 37°C for overnight and then inoculated into the 0.5 L or 1 L of LB medium supplemented with 100 μg/ml ampicillin, respectively. MazEpp and MazFpp were induced by the addition of 1 mM isopropyl β-D-1-thiogalactopyranoside when OD600 reached 2.0 and 4.0, respectively. After 3.5 hours of incubation, cells were harvested by centrifugation at 7,000 *g* and then stored at -80°C for future use.

### MazEpp Purification

*E*. *coli* cells containing MazEpp were thawed on ice and resuspended in lysis buffer (100 mM sodium phosphate buffer, 300 mM NaCl, 2.5% glycerol, 3.2 mM 2-mercaptoethanol, and 10 mM imidazole). Cells were lysed by sonication for 15 minutes with a Handy Sonic UR-20P (Tomy Seiko, Tokyo, Japan). The supernatant containing MazEpp was collected by centrifugation at 7,000 *g* for 15 minutes. Ni-NTA agarose (Qiagen) was added to the supernatant and rotated for 2 hours at 4°C to allow MazEpp to adsorb onto the Ni-NTA column. Non-specific proteins were eliminated with washing buffer (90 mM sodium phosphate buffer, 770 mM NaCl, 2.3% glycerol, 2.9 mM 2-mercaptoethanol, and 20 mM imidazole) and 100 mM imidazole buffer (97.5 mM sodium phosphate buffer, 290 mM NaCl, 2.4% glycerol, and 100 mM imidazole). MazEpp was eluted with elution buffer (92.5 mM sodium phosphate buffer, 280 mM NaCl, 2.3% glycerol, and 300 mM imidazole). The molecular weight and purity were confirmed by SDS-PAGE. Protein concentration was determined using Qubit Protein Assay Kit (Life Technologies).

### MazFpp Purification

*E*. *coli* cells containing MazFpp were thawed on ice and resuspended in binding buffer (20 mM sodium phosphate buffer, 50 mM imidazole, 300 mM NaCl, and 5 mM 2-mercaptoethanol). Suspended cells were sonicated for 15 minutes with a Handy Sonic UR-20P and then centrifuged at 7,000 *g* for 15 minutes. The supernatant was then filtered with a 0.45 μm membrane (Millex, Darmstadt, Germany). After the 1 mL His-Trap HP column (GE Healthcare, Waukesha, WI, USA) was equilibrated with 10 column volumes (cv) of binding buffer, the filtered supernatant was applied to this column and washed with 32 cv of binding buffer. Residual non-specific proteins and hexa-histidine tagged MazFpp were separated by gradually increasing the imidazole concentration using AKTA pure plus (GE Healthcare). The molecular weight and purity were confirmed by SDS-PAGE. Protein concentration was determined using Qubit Protein Assay Kit.

### RNA Preparation

The transcript of *graA* and six artificial synthetic RNAs named 500–2, 1000–1, 1000–2, 1000–3, 1000–4, and 1000–5 [33] were used in this study. All synthetic RNAs had 500-nucleotide (500–2) or 1000-nucleotide (1000–1 to 1000–5) diverse sequences [33], and each had between three-guanine bases at the 5′ end and 30-base polyadenylated tail at the 3′ end. The pMD19 plasmid encoding the *graA* gene was digested with *Hin*dIII (New England Biolabs, Ipswich, MA). pUC19 plasmids encoding synthetic RNA (500–2, 1000–1, 1000–2, 1000–3, and 1000–4) and the pUC19 plasmid encoding 1000–5 synthetic RNA were digested with *Bbs*I and *Btg*ZI, respectively (New England Biolabs). Digested fragments were purified with a PCR purification kit and then *in vitro* transcription was carried out with MEGAscript T7 Kit (Life Technologies) according to the manufacturer’s instruction. Transcribed RNAs were purified with RNA Clean & Concentrator™-5 (Zymo Research, Orange, CA, USA) and their concentration was determined using Qubit RNA Assay Kit (Life Technologies).

### Massive Parallel Sequencing

Substrate RNA was prepared by mixing 300 ng of RNA 1000–1, 1000–2, 1000–3, 1000–4, and 1000–5. This substrate was incubated at 37°C for 15 minutes with two distinct RNA interferases: 2 U of MazF (Takara) in MazF buffer (Takara) containing 4 U of recombinant RNase inhibitor (Takara) or 300 ng of MazFpp in MazFpp buffer (20 mM Tris-HCl (pH 8.0), 1 mM dithiothreitol, 0.01% tritonX-100, and 4 U of recombinant RNase inhibitor). As the control experiment, the substrate RNA was also treated with 2.5 U of RNase III (New England Biolabs) in NEBNext RNase III Reaction Buffer (New England Biolabs) at 37°C for 15 minutes. These samples were cleaned with RNA Clean & Concentrator™-5 and then incubated with 20 U of T4 polynucleotide kinase (Takara) in T4 Polynucleotide Kinase Buffer (Takara) containing 1 mM ATP (Ambion, Austin, TX, USA) at 37°C for 1 hour. They were purified using RNA Clean & Concentrator™-5. Subsequently, 125 pmol of barcode RNA and purified RNA fragments were incubated with 50 U of T4 RNA ligase (Takara) in the RNA ligation buffer (Takara) for 18 hours at 15°C. Samples were purified with RNA Clean & Concentrator™-5 and then the RNA concentration was determined using the Qubit RNA Assay Kit. Sequencing library was constructed according to the NEB Ultra RNA Library Prep Kit for Illumina protocols (New England Biolabs). For this study, the protocol for longer size RNA inserts was used. The constructed library quality was validated using the Agilent High Sensitivity DNA Kit (Agilent Technologies, Santa Clara, CA, USA). Sequencing was performed using the MiSeq platform with the MiSeq 500 cycles reagent kit v2 (Illumina, San Diego, CA, USA) according to the manufacturer’s protocol.

### Cleavage Sequence Identification

The output files configured in fastaq format for R1 and R2 reads were separately analyzed with CLC Genomics Workbench version 7.5.1 (CLC bio, Aarhus, Denmark). Nucleotides with low quality or ambiguity were eliminated with the parameters limit of 0.05 and a maximum number of ambiguities equals to zero. Reads that included the 15-base sequence (CTGGCTTTGATGAAA) corresponding to the 3′-end sequence of the barcode were extracted from both strands via the following parameters: mismatch cost and gap cost equal five, while minimum scores for internal match and end match both equal 15. All the 5′ nucleotides upstream of this 15-base sequence were removed and the reads shorter than 15 bases were discarded. The resulting reads were mapped against five references (1000–1, 1000–2, 1000–3, 1000–4, and 1000–5). The parameters used for mapping were as follows: mismatch cost, insertion cost, and deletion cost equal three; length and similarity fraction both equal one. The fastaq files were deposited into the DNA Data Bank of Japan Sequence Read Archive (DRA004282).

### Enzymatic Activity of MazFpp and MazEpp

Purified MazFpp was incubated with RNA 500–2. Next, 3 pmol of MazFpp was pre-incubated with 0.3, 3, or 30 pmol of MazEpp at room temperature for 10 minutes. After the pre-incubation, 100 ng of RNA 500–2 was added and the mix was incubated at 37°C for 30 minutes in MazFpp buffer (20 mM Tris-HCl (pH 8.0), 1 mM dithiothreitol, 0.01% tritonX-100, and 4 U of recombinant RNase inhibitor). Samples were purified by RNA Clean & Concentrator™-5 and the gel loading buffer II (Ambion) was added to each sample. Samples were incubated at 95°C for 5 minutes and separated on a 10% polyacrylamide gel containing 7 M urea. RNA was stained using SYBR Gold (Life Technologies) and then detected with a Typhoon 9210 imager (GE Healthcare).

### Fluorometric Detection of MazFpp Activity

A fluorometric assay [34] was used to validate the cleavage sequences. One hundred nanograms of MazFpp or RNase A (Novagen) and 20 pmol of fluorescent-labeled oligonucleotides were incubated in MazFpp buffer (20 mM Tris-HCl (pH 8.0), 1 mM dithiothreitol, 0.01% tritonX-100, and 4 U of recombinant RNase inhibitor). All reactions were conducted at 37°C in triplicate and fluorescent intensity was recorded every 60 seconds using a Light Cycler 480 system (Roche, Basel, Switzerland) with 483 nm excitation and 533 nm detection filters.

### RNA Cleavage Specificity of MazFpp

One hundred nanograms of *graA* transcript and RNA 500–2 were incubated with 50 ng of MazFpp at 37°C in MazFpp buffer (20 mM Tris-HCl (pH 8.0), 1 mM dithiothreitol, 0.01% tritonX-100, and 4 U of recombinant RNase inhibitor). After incubation for 1, 5, 15, or 30 minutes, gel loading buffer II was added to each sample. Samples were incubated at 95°C for 5 minutes before electrophoresis on a 10% polyacrylamide gel containing 7 M urea. RNA was then stained with SYBR Gold and detected with a Typhoon 9210 imager.

### Accession Numbers and GI Numbers

The GenBank accession numbers and GI numbers were as follows: *mazEpp* gene (NC_002947.3, 26986745), *mazFpp* gene (NC_002947.3, 26986745), *graA* gene (AE015451.1, 24987239), 500–2 (AB610940.1, 321117288), 1000–1 (AB610944.1, 321117292), 1000–2 (AB610945.1, 321117293), 1000–3 (AB610946.1, 321117294), 1000–4 (AB610947.1, 321117295), and 1000–5 (AB610948.1, 321117296).

## Results

### Massive Parallel Sequencing Identified the MazF Cleavage Sequence

In massive parallel sequencing, nucleotides are sequenced with significant depth, which generates reliable data about the frequency of the detected molecules [35]. Indeed, this sequence depth was critical for identifying the RNA cleavage sites using a high-throughput approach. We used five 1000-nt synthetic RNAs designed by a computer algorithm as the substrates for RNA interferases. These RNAs are well suited to this approach because they are long enough and they are composed of nearly equal numbers of each RNA base (A, U, G, and C) [33]. This increases the possibility that the cleavage sequence will be included in the RNAs. Another advantage of these RNAs is that they were designed in a way that they do not form complex secondary structures [33]. Because of this smart design, RNA unwinding proteins like CspA, which prevent secondary-structure formation of substrate RNA and enhances the accessibility of RNA interferases to their cleavage sites [22,26,27], were not required for the experiment.

**Fig 1:**
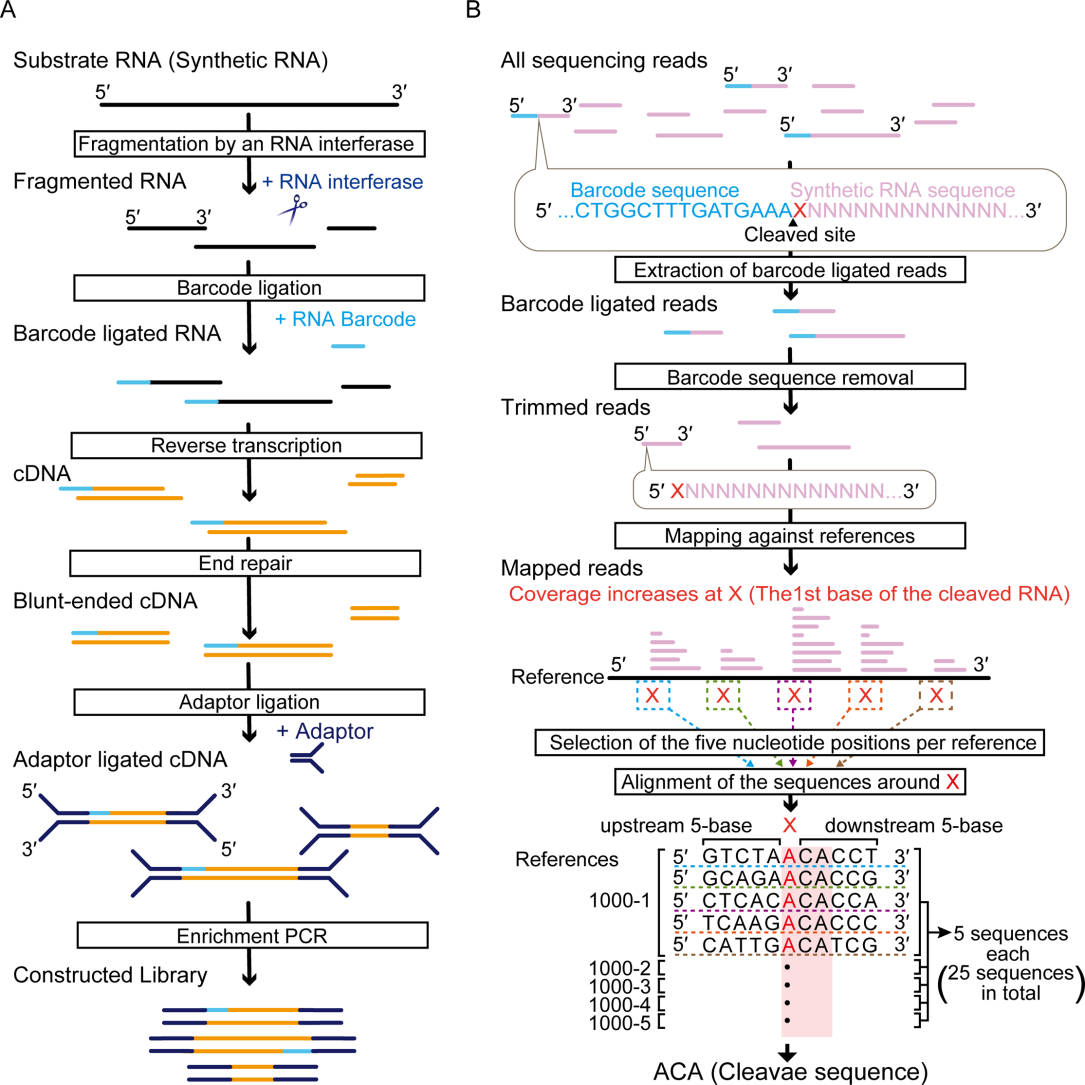
Schematic representation of the experimental methods. (A) Overview of the sequence library construction method. (B) Workflow for identification of RNA cleavage site (see text).

MazF is one of the best-characterized RNA interferases, and it was originally identified in an *Escherichia coli* chromosome [4]. Since the ACA triplet in single-stranded RNA was already elucidated as its cleavage sequence, we chose *E*. *coli* MazF as a model endoribonuclease and then evaluated if our approach was consistent with previous results [12,36]. To this end, using the Illumina platform we examined the 5′-end sequences of the MazF fragmented RNAs. A challenge of this approach was that the sequence information for the cleaved RNA sites was eliminated during the procedure in order to construct the sequence library: single-stranded DNA overhangs were polished in the end repair step. To overcome this challenge and to maintain the 5′-end nucleotides derived from the original RNA strand, which would retain the MazF cleaved sequences, we ligated a 45-nt RNA barcode oligonucleotide whose sequence was identical to the 5′ RACE Adapter of the FirstChoice RLM-RACE Kit (S1 Table, Fig 1A).

**Fig 2:**
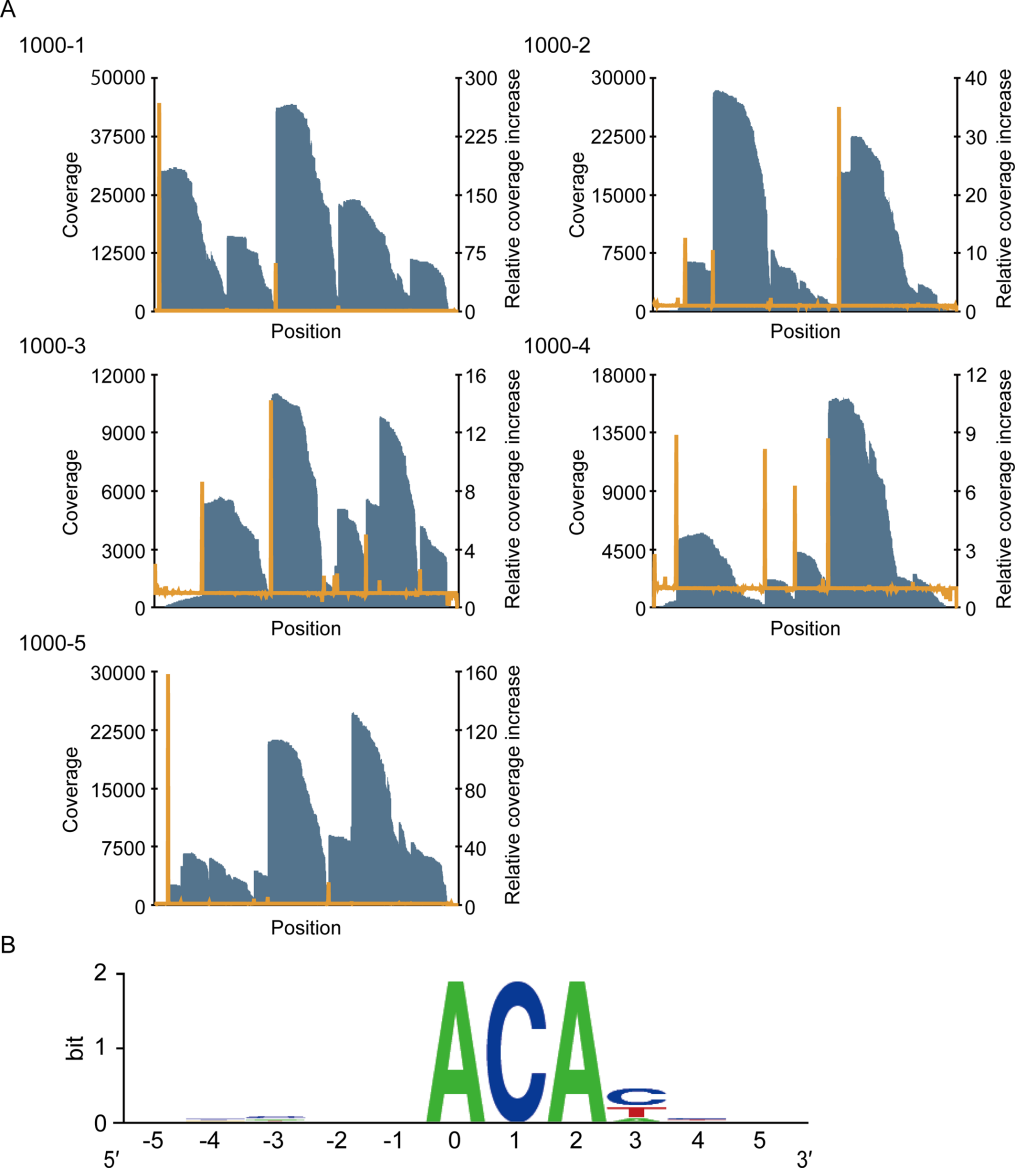
Identification of the MazF cleavage sequence using massive parallel sequencing. (A) Graph of the coverage (blue bar) and the relative coverage increase (orange line). (B) Graphical representation of the conserved sequences. The nucleotide position with significant increases in coverage was numbered as zero. Twenty-five sequences were analyzed (S2 Table) and the frequency at each position was visualized with the WebLogo program [37].

In our approach, the 5′-end sequence of the fragmented RNAs immediately downstream of the 3′-end sequence of the barcode is considered to be identical with the MazF cleavage sequence (Fig 1B). We extracted the reads containing the 15-base sequence (CTGGCTTTGATGAAA) corresponding to the 3′-end sequence of the barcode and then removed all the 5′-end nucleotides including the 15 bases from the extracted reads. These trimmed reads were mapped against the five reference sequences (1000–1, 1000–2, 1000–3, 1000–4, and 1000–5) (Fig 1B), resulting in the recovery of 9.21% of total reads. The reason for this low recovery was considered to be the low efficiency of the barcode ligation, as most of the reads discarded in the analysis procedures were derived from non-ligated RNA molecules. In all reference sequences, we found increases in coverage at specific nucleotides (Fig 2A). The sequence adjacent to these nucleotides includes the cleavage sequences. To identify the cleavage sequence, we searched for the nucleotides that met the following two criteria (X in Fig 1B): first, the position showed the coverage over 1000; and second, the positions had a large relative coverage increase, which is the value defined as the coverage at the n+1^th^ position divided by the coverage at the n^th^ position. The relative coverage increase at each nucleotide position was arranged in decreasing order in each reference, and five nucleotides that showed the five largest relative coverage increases were selected from all references (S2 Table). We extracted the sequences including five bases upstream and five bases downstream (Fig 1B). When next aligned 25 sequences (five sequences from five references) (Fig 1B, S2 Table), we found ACA was correctly highlighted as the MazF cleavage sequence (Fig 2B). Importantly, no specific sequence was detected in a control experiment that used RNase III, a structure-specific endoribonuclease (S1 Fig, S3 Table). These results prove that our approach is useful for detection of RNA interferase-specific cleavage sequences.

### MazFpp Is a Bona-Fide RNA Interferase and Comprises a TA System with MazEpp

Having determined the MazF cleavage sequence as ACA, we next tried to characterize the RNA interferase with unknown cleavage specificity. For this purpose, we chose a putative MazF homologue encoded by the locus PP0771 in *Pseudomonas putida* chromosome (hereafter MazFpp). Since this bacterium is widely used for production of chemical compounds and it is also a xenobiotic decomposer in environmental engineering [38], characterization of the toxin enzyme that regulates its growth would be beneficial to understand its industrial utility.

**Fig 3:**
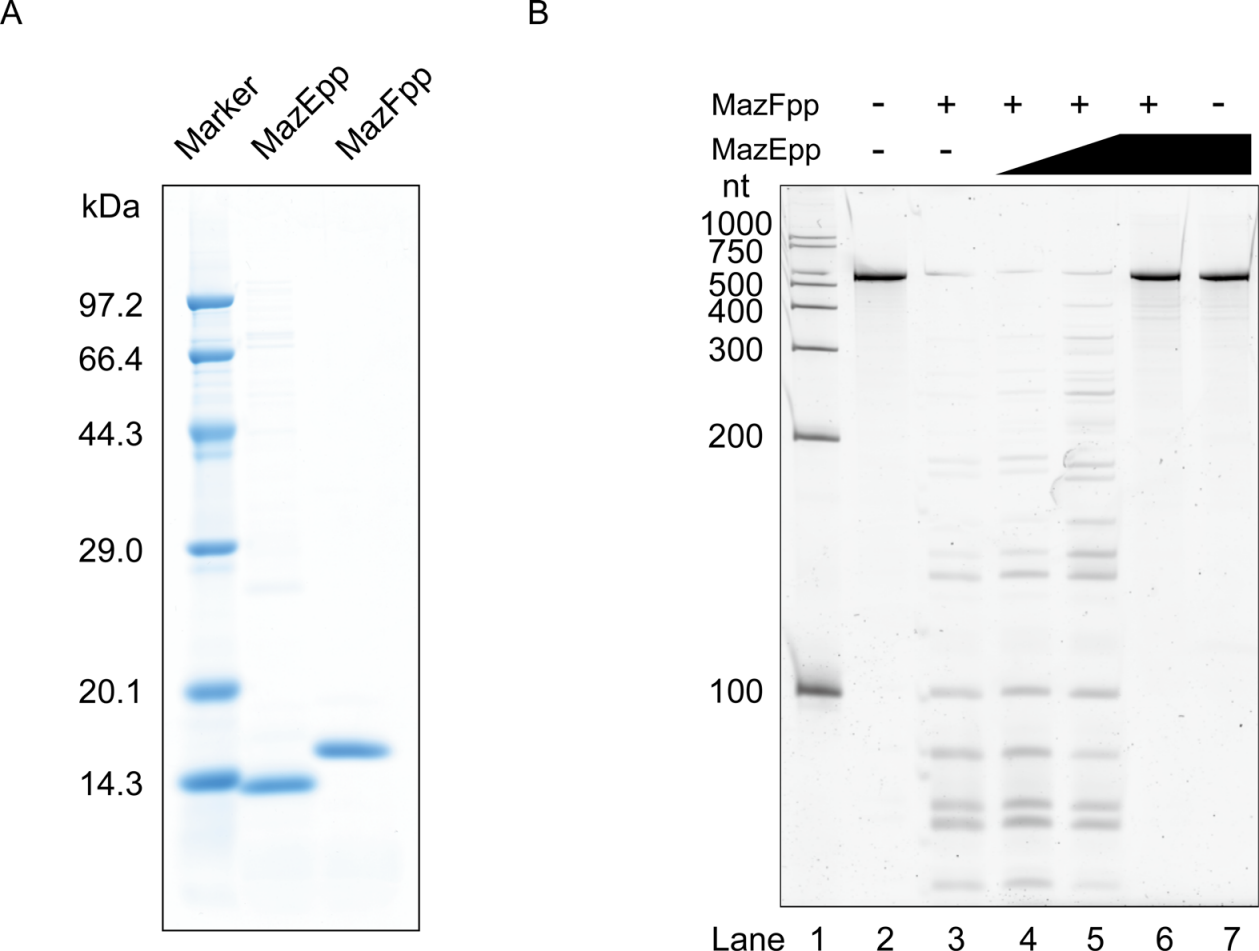
MazEF pairs in *Pseudomonas putida*. (A) Molecular weight and the purity of the hexa-histidine tagged MazEpp and MazFpp. (B) Enzymatic activity of MazEpp and MazFpp. A 533-base synthetic RNA (RNA 500–2) was incubated with these enzymes. Lane 2, a control reaction without any enzymes; Lanes 3–6, 3 pmol of MazFpp was added. For lanes 4–6, MazE-pp was also added to each lane: 0.3, 3, and 30 pmol respectively. Lane 7, 30 pmol of MazEpp was added.

Though MazFpp shows 34.8% identity to *E*. *coli* MazF, there is no evidence of its function. Hence, we isolated histidine-tagged MazFpp (Fig 3A) and then incubated it with an RNA substrate to examine if MazFpp possesses endoribonuclease activity. Several short fragments were observed when substrate RNA was treated with MazFpp, indicating that MazFpp indeed functions as an RNA interferase and recognizes relatively short sequences (Fig 3B, Lane 3). In order to rule out the possibility that this cleavage was due to the contaminated RNases, we next purified a cognate antitoxin MazEpp (PP_0770) (Fig 3A). Following the pre-incubation with MazEpp, we observed that the ribonuclease activity of MazFpp was inhibited in a dose-dependent manner (Fig 3B, Lanes 4–6). On the basis of these findings, we concluded that MazFpp is a bona-fide RNA interferase and constitutes a TA system together with MazEpp.

### MazFpp Specifically Recognizes UAC Triplet

**Fig 4:**
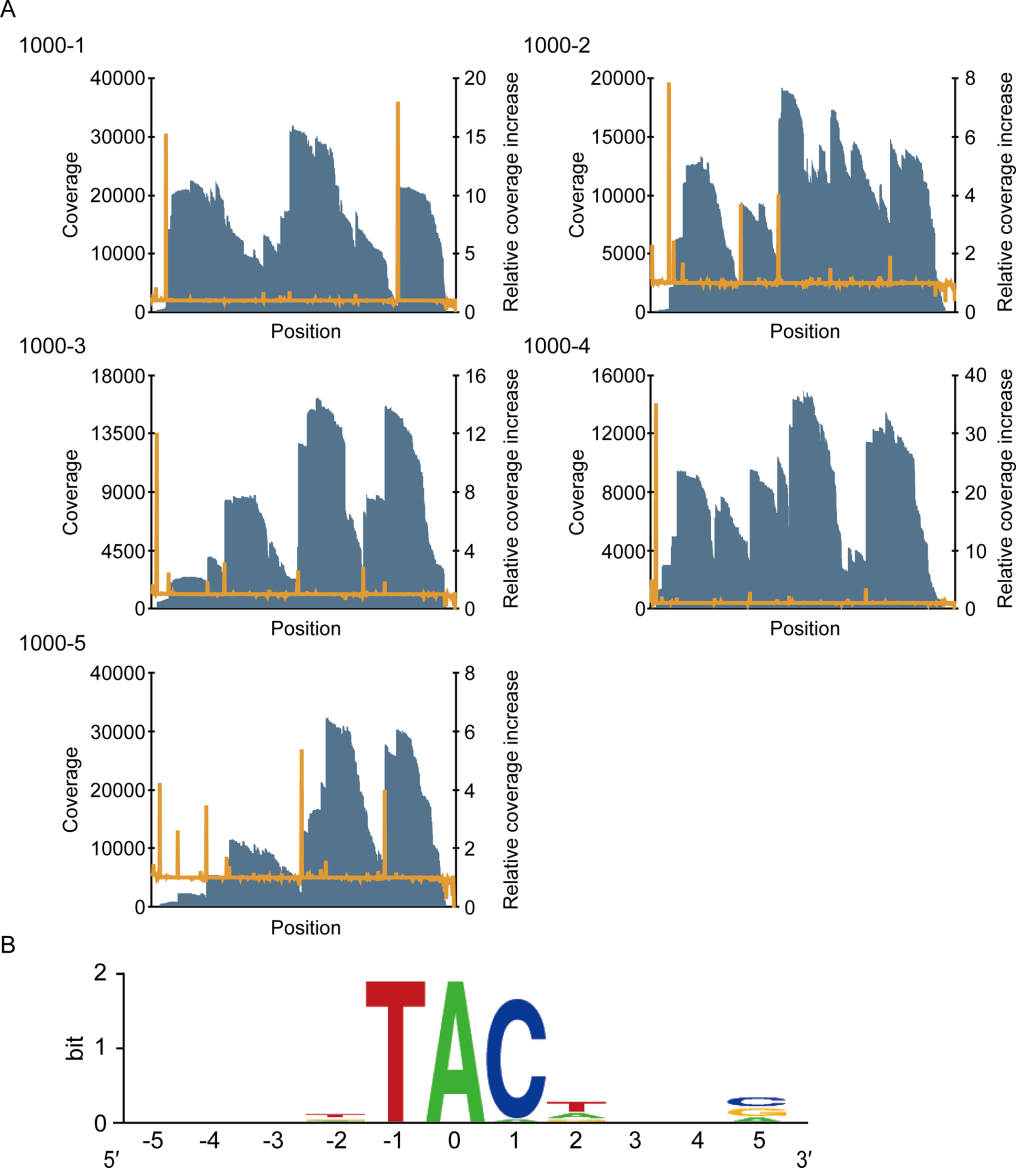
Analysis of the cleavage sequence of MazFpp. (A) Graph of the coverage (blue bar) and the relative coverage increase (orange line). (B) The conserved sequence around nucleotide positions with increased coverage. The nucleotide position with significant increases in coverage was set to zero.

To determine the cleavage specificity of MazFpp, the MazFpp cleaved sites within the five synthetic RNAs were analyzed using massive parallel sequencing in the same manner as for *E*.*coli* MazF (Fig 4A). When the 25 sequences were aligned, the UAC triplet was highlighted as a potential cleavage sequence. Since the coverage increased at the adenine, we speculated that MazFpp specifically recognizes a UAC triplet and cuts the RNA between U and A (S4 Table, Fig 4B).

**Fig 5:**
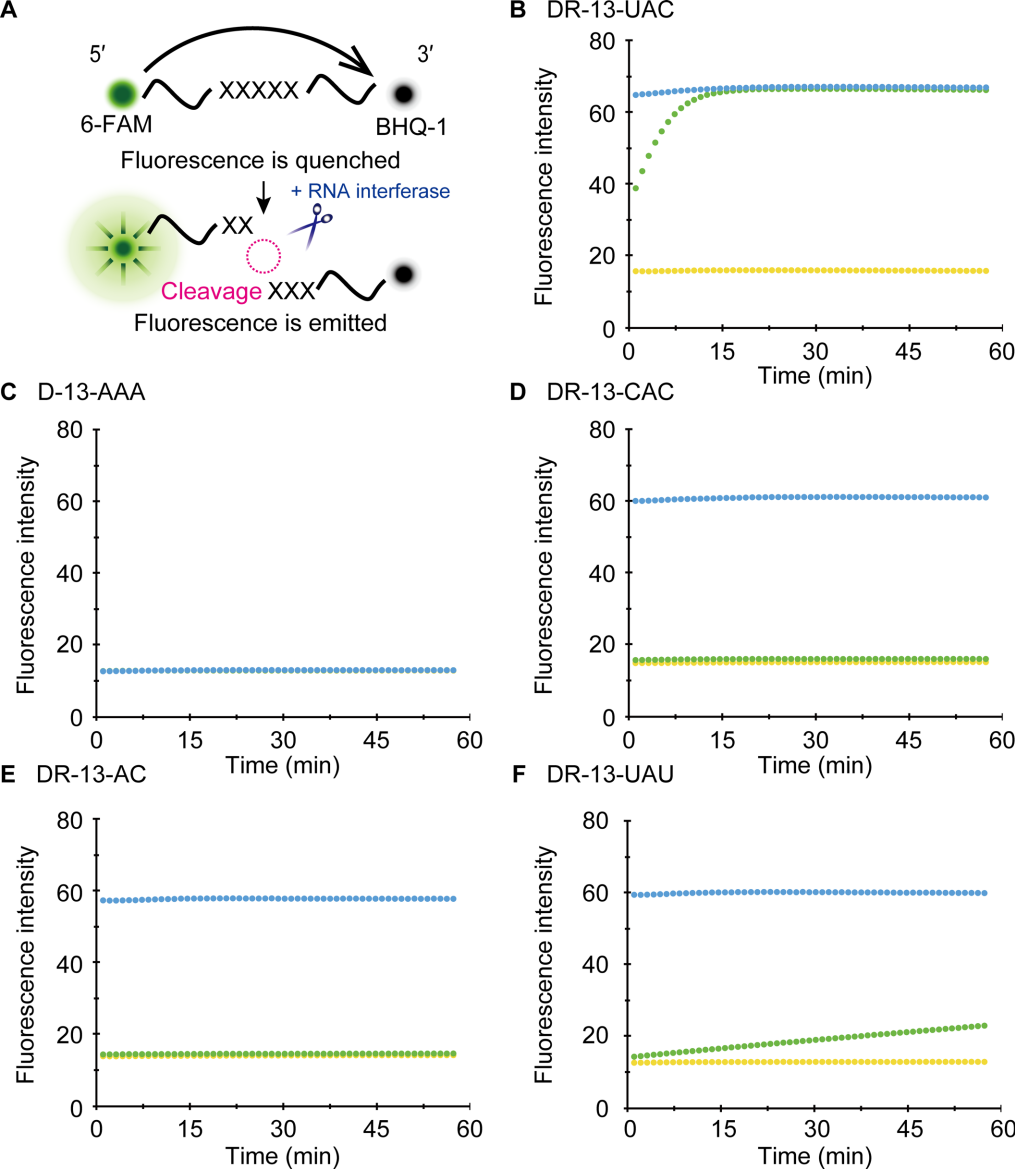
Fluorometric detection. (A) Principle of the fluorometric detection based on the fluorescence resonance energy transfer [34]. The fluorescent of 6-FAM is typically quenched by BHQ-1, but it emits a bright signal when the oligonucleotide is cleaved. (B-F) Investigation on the MazFpp cleavage site. One hundred nanograms of RNase A (blue) or MazFpp (green) was incubated with each oligonucleotide. The yellow plot represents a control reaction in which no enzyme was added.

We next used a fluorometric assay [34] to further validate our results. In this assay, oligonucleotides were tagged with two fluorescent dyes, 6-carboxyfluorescein (6-FAM) as a reporter and black hole quencher-1 (BHQ-1) as a quencher, and then they were incubated with MazFpp. Fluorescence intensity was continuously measured. Since 6-FAM and BHQ-1 are in close proximity, 6-FAM is normally quenched. When MazFpp cleaves the oligonucleotides, however, these two dyes are spatially separated, resulting in a gradual increase of the fluorescence (Fig 5A). We designed various 13-base DNA, RNA, and DNA/RNA chimeric oligonucleotides (Table 1) and sought to verify the cleavage specificity of MazFpp.

**Table 1:** Fluorescent-labeled oligonucleotides used in fluorometric assay.

Name	Sequence (5′ to 3′) ^a^
DR-13-UAC	AAAAAUACAAAAA
D-13-AAA	AAAAAAAAAAAAA
R-13-UCUCG	UCUCGGUGCGUUG
R-13-GUUGU	GUUGUCAUGCCGG
R-13-UGACA	UGACACGAACCGC
DR-13-CAC	AAAAACACAAAAA
DR-13-AC	AAAAATACAAAAA
DR-13-UAU	AAAAAUAUAAAAA

^a^ Underlined letters represent RNA nucleotides and the other letters represent DNA nucleotides.

In accordance with the RNA-seq results, when a DNA/RNA chimeric oligonucleotide, DR-13-UAC, was treated with MazFpp, fluorescence intensity rapidly increased to values similar to the RNase A treated oligonucleotides (Fig 5B). In contrast, D-13-AAA, which is composed of a 13-base DNA adenine repeat, was not digested (Fig 5C). No cleavages were observed with three other RNA oligonucleotides (R-13-UCUCG, R-13-GUUGU, and R-13-UGACA) whose sequences are present in one of the synthetic RNA constructs (RNA 1000–4) used as a substrate for massive parallel sequencing (S2 Fig). As they did not include any UAC sequences, these results reinforced the notion that MazFpp shows endoribonuclease activity specifically against the UAC triplet.

We prepared three additional fluorogenic oligonucleotides with single base replacement (DR-13-CAC, DR-13-AC, DR-13-UAU). As we anticipated, substitution of the first U to the other pyrimidine base C or T (DR-13-CAC or DR-13-AC) completely impaired the MazFpp-mediated cleavage (Fig 5D and 5E). However, with DR-13-UAU, slight increase in fluorescent intensity was observed (Fig 5F). Taken together, MazFpp cleaves the UAC triplet between U and A, but also weakly recognizes other specific sequences. In addition, since the replacement of U to T completely blocked activity (DR-13-AC) (Fig 5E), the 2′ OH of the pentose [36,39] and/or the presence of a methyl group are important for the RNA cleavage. In contrast to the RNA 500–2, the transcript lacking UAC triplets (*graA* transcript in *P*. *putida* genome) was indeed stable even in the presence of MazFpp (S3 Fig). Taken together, these results demonstrated that the UAC sequence is the main target of this RNA interferase.

## Discussion

RNA interferases, which compose TA systems, are the toxin endoribonucleases that disrupt the stability of intracellular RNAs by cleaving them in a ribosome independent [12–16] or dependent [7–11] manner. Intriguingly, some RNA interferase families such as VapC, MqsR, and MazF were reported to cleave the RNA molecules (tRNA, mRNA, and/or rRNA) in a sequence-specific manner [12,13,20,22–27,32,40–42] and to be implicated in microbial stress adaptation by regulating the translation patterns [28,29,43–46]. However, despite their widespread representation in microbial genomes, often with multiple loci [2,3,21], their sequence-specificities are poorly understood due to limitations of conventional methods for cleavage sequence detection [22,26,27,30].

In this study, we developed an easy-to-use method to define the cleavage pattern of RNA interferases with the Illumina platform (Fig 1). This method correctly identified the requisite sequence for the *E*. *coli* MazF cleavage as ACA (Fig 2). One unanticipated finding was that of the two different, previously identified positions of the cleavage site of *E*. *coli* MazF (^ACA or A^CA, where ^ is the position of the cleavage sites) [36], only ^ACA was detected in our analysis, suggesting that this endoribonuclease preferably cleaves the designed RNAs at the 5′ end of ACA. Furthermore, it successively revealed that *P*. *putida* MazF could cleave RNA at UAC triplets (Fig 4). Notably, the sequence identity between MazFpp and MazF-mt1 (Rv2801c), a MazF endoribonuclease conserved in *Mycobacterium tuberculosis* [47], is low (28.8%), despite the fact that both RNA interferases specifically recognize the UAC triplet. VapC_Rv0065_ and VapC_Rv0617_ only share 22% identity, but recognize the same 4-nt motif [30]. Similarly, MazFpp and MazF-mt1 recognize the same sequence, but the reasons for this remain unclear.

The five 1033 nucleotide synthetic RNAs employed in this study also facilitated this detection. When compared to the MORE RNA-seq, the sequence diversity of these RNAs was not rich, and thus, our method is not suitable for characterizing long base cutters such as MazF-hw, which recognizes seven bases in a strict manner [26]; indeed, these RNAs cover only 25.6% of the possible heptads (S5 Table). However, they still cover a variety of triplets, quartets, and pentads (S6 Table): 100%, 98.4%, and 95.5%, respectively. Moreover, it is feasible to design several other RNAs, which would further improve the accuracy of the cleavage-specificity determination. Based on previous reports stating that most of the RNA interferases specifically recognize less than or equal to five bases [12–14,22–24,27,41,48], these sequence combinations would be helpful for identifying which RNAs are targets in microbial cells in many cases. The notion that pathogenic bacteria and autotrophic microorganisms encode multiple RNA interferases is now widely accepted [2]. Therefore, an effective strategy to study their physiological functions would be to induce RNA interferases that are indispensable for stress adaptation, and then characterize them first with whole transcriptome and/or proteome analysis followed by our approach. A possible roadblock of our approach is that some bases might be counted as a potential cleavage sequence. For example, some might speculate that C or T at position 3 in Fig 2B and T at position 2 in Fig 4B are also involved in cleavage. The possibilities are negated with fluorometric analysis from fluorescence resonance energy transfer experiments [34], because 6-FAM-mediated intense signal was only detected when the oligonucleotide is cleaved by the RNA interferase (Fig 5 and S2 Fig).

It is known that transcripts lacking recognition sequences are not cleaved in cells [22,24,26,32]. Therefore, we attempted to identify transcripts that are translated under the environments where MazFpp is liberated. To this end, we searched for all 5350 coding sequences predicted in the *P*. *putida* genome and we found that 97 coding sequences lack UAC triplets. Interestingly, UAC triplets were also absent in the transcript of the antitoxin gene, encoded by locus PP1585. Recently, Tamman et al. demonstrated that this antitoxin composes a genuine TA pair that belongs to the HigBA family, and they named this TA pair GraTA (Growth rate-affecting toxin-antitoxin), consisting of GraT toxin and GraA antitoxin [49]. In this report, the *graTA* operon was efficiently repressed by GraA antitoxin, probably via its DNA binding domain. Since the *graA* transcript is tolerant of MazFpp (S3 Fig), the expression of GraT, which inhibits microbial growth at low temperatures but improves tolerance against some antibiotics [49], might also be negatively regulated by the preferentially translated GraA when MazFpp is released in cells. Therefore, *P*. *putida* might use these toxins selectively, depending on the environmental stresses. Moreover, since *P*. *putida* GraT has 37% sequence similarity to *Vibrio chrolerae* HigB, an RNA interferase that also mediates mRNA cleavage [49,50], GraT might also destabilize some intracellular RNAs. In that case, *P*. *putida* presumably improves the resistance against specific stresses by utilizing RNAs that elude the MazFpp or GraT-mediated cleavage.

In this report, we described a novel method to analyze RNA interferases with massive parallel sequencing. This approach is not specific to the MazF family of endoribonucleases but all RNA interferases that cleave intracellular RNAs in a sequence-specific manner. Furthermore, it is compatible with the fluorometric assays, enabling an easy and accurate determination of cleavage sequences in a high-throughput manner. Our approach would be useful to investigate the majority of uncharacterized RNA interferases that are conserved in the prokaryotic domain. This would further expand our understanding about physiological functions of RNA interferases.

## Supporting Information

S1 Fig Application of the massive parallel sequencing to a structure-specific endoribonuclease. (A) Graph of the coverage (blue bar) and relative coverage increase (orange line). (B) Graphical representation of the sequences around the base with increases in coverage. The nucleotide position with significant increases in coverage was numbered as zero. Twenty-five sequences were analyzed (S3 Table) and the frequency at each position was visualized with the WebLogo program.
(TIF)

S2 Fig Fluorometric detection [34]. One hundred nanograms of RNase A (blue) or MazFpp (green) was incubated with RNA oligonucleotides. The yellow plot represents a control reaction where no enzyme was added.
(TIF)

S3 Fig Cleavage specificity of MazEpp. Synthetic RNA 500–2 or *graA* transcript, which do or do not include UAC triplets, respectively, were incubated with MazFpp. Lanes 2 and 8, control reactions in which no enzyme was added; Lanes 3–6, 100 ng of MazFpp was incubated with RNA 500–2; Lanes 9–12, 100 ng of MazFpp was incubated with *graA* transcript.
(TIF)

S1 Table Sequence of PCR primers and the barcode RNA used in this study. (PDF)

S2 Table Extracted 25 sequences with MazF cleavage. (PDF)

S3 Table Extracted 25 sequences with RNase III fragmentation. (PDF)

S4 Table Extracted 25 sequences with MazFpp cleavage. (PDF)

S5 Table Number of occurrences of heptads in the substrates. (PDF)

S6 Table Number of occurrences of pentads in the substrates. (PDF)
