# Supplementary figures and images for: Characterization of MazF-Mediated Sequence-Specific RNA Cleavage in Pseudomonas putida Using Massive Parallel Sequencing
Source: PLoS One. 2016 Feb 17;11(2):e0149494. doi: 10.1371/journal.pone.0149494 (PMC4757574; doi:10.1371/journal.pone.0149494)

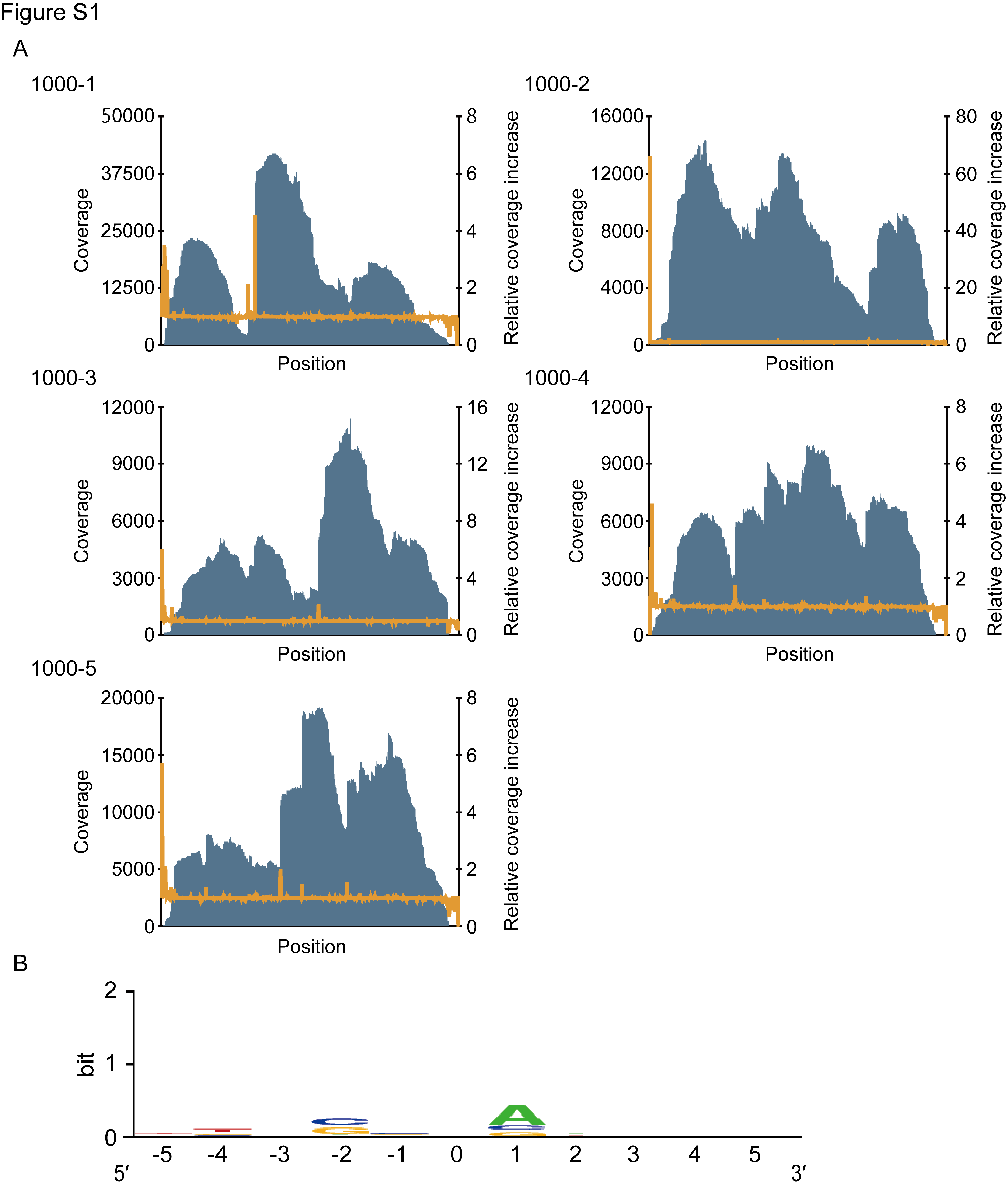

Supplement: S1 Fig — (A) Graph of the coverage (blue bar) and relative coverage increase (orange line). (B) Graphical representation of the sequences around the base with increases in coverage. The nucleotide position with significant increases in coverage was numbered as zero. Twenty-five sequences were analyzed (S3 Table) and the frequency at each position was visualized with the WebLogo program. (TIF) [file pone.0149494.s001.tif]

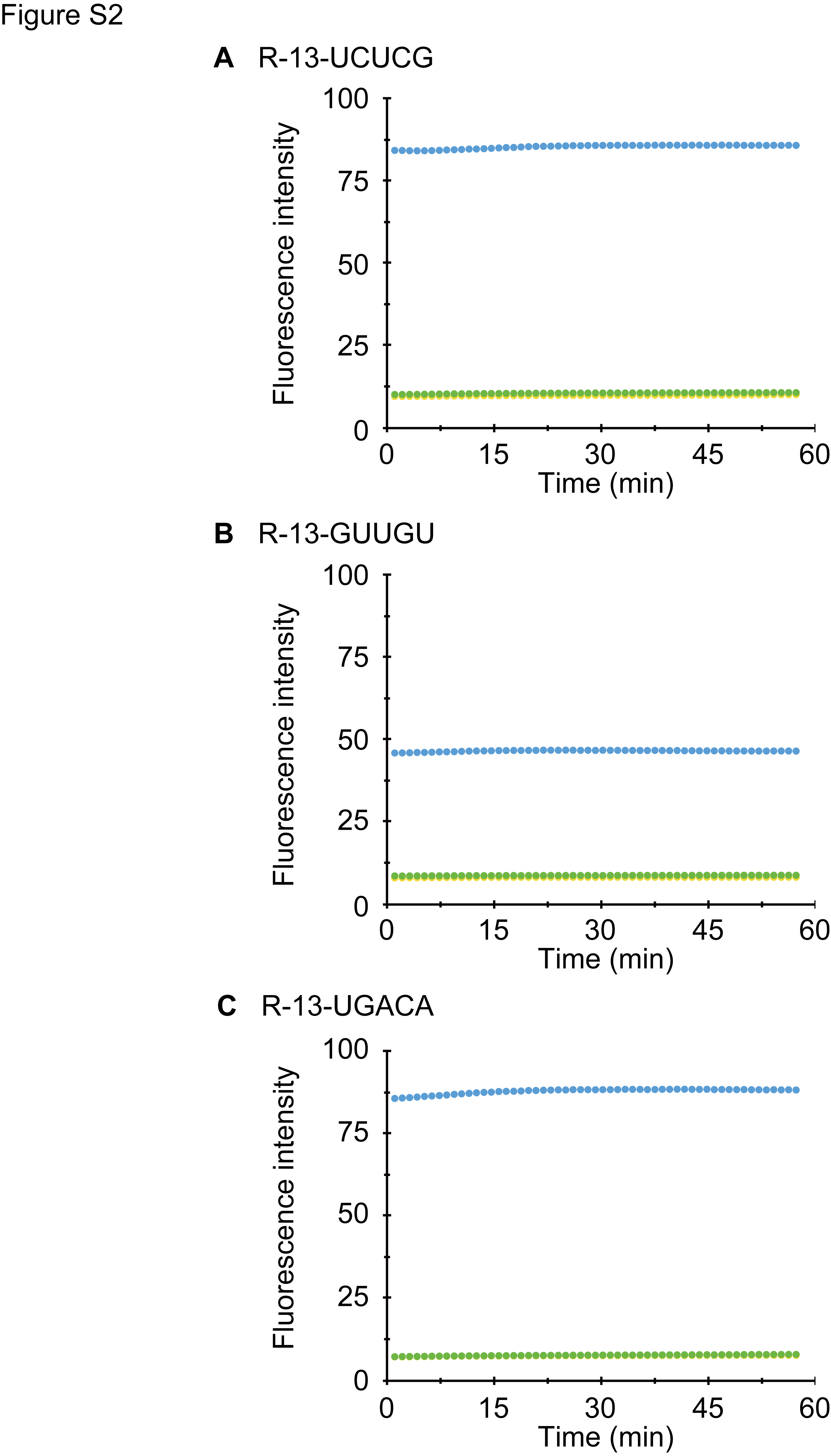

Supplement: S2 Fig — One hundred nanograms of RNase A (blue) or MazFpp (green) was incubated with RNA oligonucleotides. The yellow plot represents a control reaction where no enzyme was added. (TIF) [file pone.0149494.s002.tif]

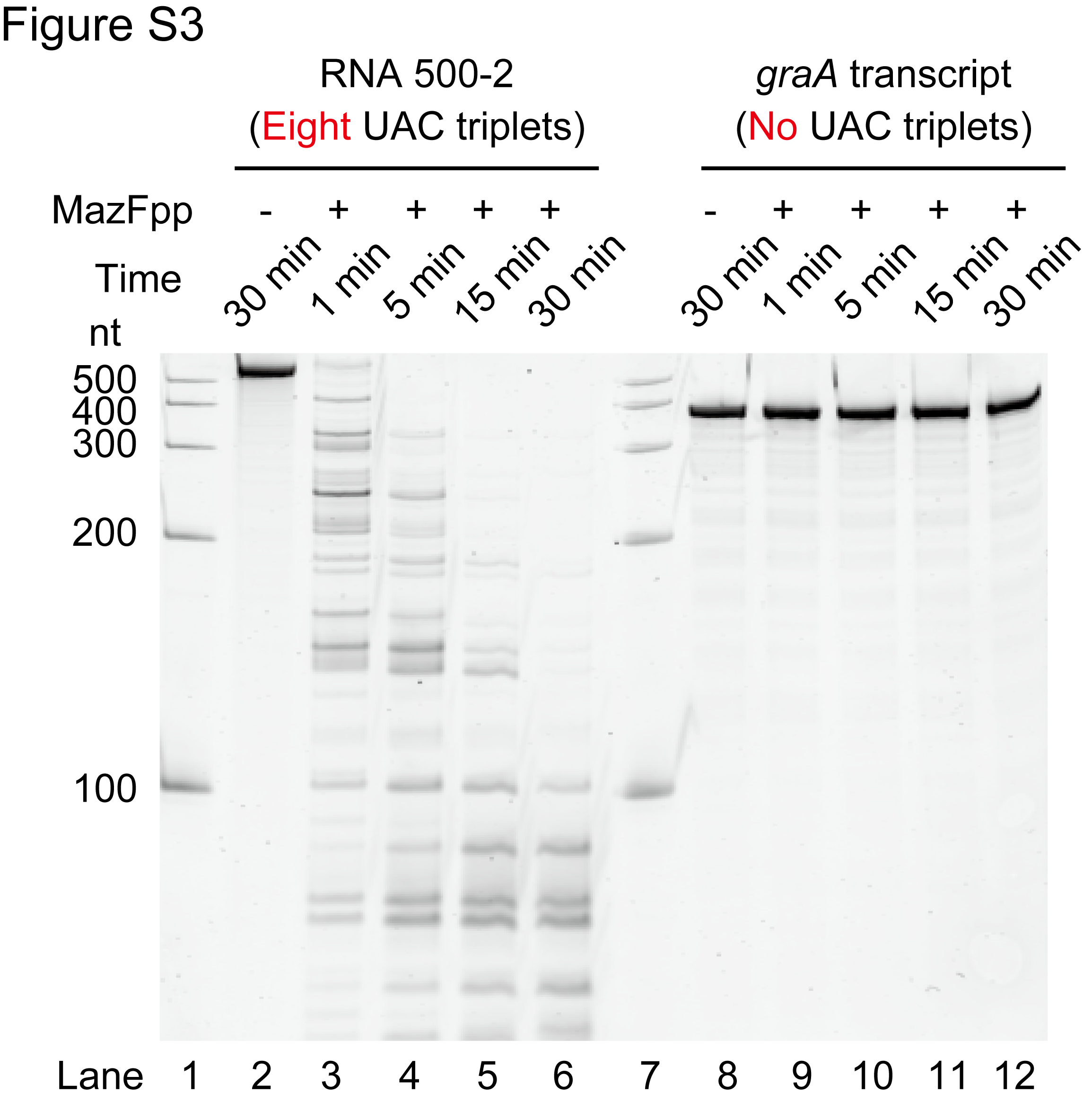

Supplement: S3 Fig — Synthetic RNA 500–2 or graA transcript, which do or do not include UAC triplets, respectively, were incubated with MazFpp. Lanes 2 and 8, control reactions in which no enzyme was added; Lanes 3–6, 100 ng of MazFpp was incubated with RNA 500–2; Lanes 9–12, 100 ng of MazFpp was incubated with graA transcript. (TIF) [file pone.0149494.s003.tif]
