# Supplementary material for: Characterization of MazF-Mediated Sequence-Specific RNA Cleavage in Pseudomonas putida Using Massive Parallel Sequencing
Source: PLoS One. 2016 Feb 17;11(2):e0149494. doi: 10.1371/journal.pone.0149494 (PMC4757574; doi:10.1371/journal.pone.0149494)
Supplement: S1 Table — (PDF) [file pone.0149494.s004.pdf]

Table S1

Name	Sequence (5′ to 3′)
mazEpp-forward	GGGGATCCGGATGCAGATCAAGATTCAACAGTGG
mazEpp-reverse	GGGAATTCGGCACTTCACGCCCCACTGG
mazFpp-forward	GGGGATCCGGGTGAAACGGTTGAAATTCGCCAG
mazFpp-reverse	GGGAATTCGGATCGAATAGGGTTTGCACACG
barcode RNA	GCUGAUGGCGAUGAAUGAACACUGCGUUUGCUGGCUUUGAUGAAA
