# Supplementary material for: Characterization of MazF-Mediated Sequence-Specific RNA Cleavage in Pseudomonas putida Using Massive Parallel Sequencing
Source: PLoS One. 2016 Feb 17;11(2):e0149494. doi: 10.1371/journal.pone.0149494 (PMC4757574; doi:10.1371/journal.pone.0149494)
Supplement: S2 Table — (PDF) [file pone.0149494.s005.pdf]

Table S2

RNA type	Rank	Position	Relative coverage increase	Coverage	Sequence (5′ to 3′) [a]
1000-1	1	16	268.2	29,499	GTCTAACACCT
	2	412	62.3	42,992	GCAGAACACCG
	3	625	7.5	22,707	CTCACACACCA
	4	246	4.4	15,874	TCAAGACACCC
	5	869	3.3	11,200	CATTGACATCG
1000-2	1	632	35.1	17,683	AAAGGACACTC
	2	109	12.6	6,249	ACCATACAACC
	3	203	10.5	27,614	GCCCAACACGT
	4	400	2.3	7,877	ACCCAACATAA
	5	901	1.5	3,566	CGTTTACACCG
1000-3	1	396	14.2	10,600	TCAGGACATAC
	2	162	8.6	5,241	GGCCGACAAGT
	3	719	5.0	5,529	AGCGTACATTC
	4	903	2.6	4,187	TCTAGACAATA
	5	621	2.3	5,036	GGTAGACACCG
1000-4	1	79	8.9	5,112	GCGTCACACCT
	2	595	8.7	15,066	GGTACACACAG
	3	380	8.2	2,131	CTCTAACACTC
	4	482	6.3	4,241	TGAATACACGT
	5	577	1.5	2,390	CGCGTACATTT
1000-5	1	592	15.7	8,841	CCCATACAACC
	2	385	5.6	20,660	TAGATACACTC
	3	338	4.3	4,421	GAATAACATGC
	4	48	2.7	2,619	GGAACACATCG
	5	670	2.4	24,424	GGACAACATTC

[a] Underlined letters represent the base with significant coverage increase
