# Supplementary material for: Characterization of MazF-Mediated Sequence-Specific RNA Cleavage in Pseudomonas putida Using Massive Parallel Sequencing
Source: PLoS One. 2016 Feb 17;11(2):e0149494. doi: 10.1371/journal.pone.0149494 (PMC4757574; doi:10.1371/journal.pone.0149494)
Supplement: S3 Table — (PDF) [file pone.0149494.s006.pdf]

Table S3

RNA type	Rank	Position	Relative coverage increase	Coverage	Sequence (5′ to 3′) [a]
1000-1	1	326	4.6	35,230	TCCCTTCAGAA
	2	15	2.8	3,621	GGTCTAACACC
	3	12	2.7	1,108	ATCGGTCTAAC
	4	21	2.6	9,945	ACACCTATCGC
	5	302	2.1	5,853	TGAAGGGAACA
1000-2	1	69	2.3	5,301	TGACCGAATCC
	2	41	2.0	1,350	TTTGACGGTGC
	3	763	1.5	4,511	CTAGCGAGGCG
	4	793	1.3	7,417	CTCGCTAGACG
	5	760	1.3	2,904	CTTCTAGCGAG
1000-3	1	546	2.2	4,967	TGTCCTAAGCT
	2	518	1.3	2,552	TGCGGACTGTC
	3	72	1.3	2,301	CCTCTAATCGC
	4	322	1.3	4,677	TCGCGACTATC
	5	462	1.3	2,294	AGTTCTAAGGT
1000-4	1	299	1.8	5,684	TGTTCGATGCG
	2	752	1.4	6,519	ATCGAATACTG
	3	41	1.3	1,477	ATACGTAAGGC
	4	84	1.3	2,835	ACACCTGCTTA
	5	398	1.3	7,699	CAGGTAATAGC
1000-5	1	414	2.0	10,590	GTACCGAAGCT
	2	646	1.5	12,564	TTCGGTATACC
	3	489	1.5	17,929	GAGACCAACTA
	4	156	1.4	7,981	TTGGTCACGCT
	5	35	1.4	2,403	TTCGGAATCTT

[a] Underlined letters represent the base with significant coverage increase
