# Supplementary material for: Characterization of MazF-Mediated Sequence-Specific RNA Cleavage in Pseudomonas putida Using Massive Parallel Sequencing
Source: PLoS One. 2016 Feb 17;11(2):e0149494. doi: 10.1371/journal.pone.0149494 (PMC4757574; doi:10.1371/journal.pone.0149494)
Supplement: S4 Table — (PDF) [file pone.0149494.s007.pdf]

Table S4

RNA type	Rank	Position	Relative coverage increase	Coverage	Sequence (5′ to 3′) [a]
1000-1	1	837	18.0	21,108	CTATTACTTCG
	2	49	15.3	13,897	GATATACTGCC
	3	469	1.8	30,996	TTCTTACCAGC
	4	380	1.7	13,145	TAAGTACACGA
	5	693	1.6	17,256	GATTTACTGCC
1000-2	1	62	7.9	2078	CGGCTACTGAC
	2	434	4.0	16,347	GGCTTACAGTG
	3	306	3.7	9,344	ATGATACTAGA
	4	78	2.5	5,406	CCCTTACGTAA
	5	813	1.9	14,705	CTAGTACAGCC
1000-3	1	248	3.2	8,281	AGATTACTAGA
	2	719	2.9	7,459	AGCGTACATTC
	3	498	2.6	12,618	ATAATACGACC
	4	58	2.5	1928	GCGTTAATTAG
	5	190	1.9	3,960	GAGGTACTTAG
1000-4	1	18	35.2	1302	ACTGTACTCTC
	2	731	3.5	11,370	CGTTTACTGAC
	3	337	2.9	9,459	CCAATACGTTG
	4	470	2.2	12,505	GGAGTACTGAC
	5	38	2.1	2,849	ACCATACGTAA
1000-5	1	510	5.4	12,967	CGTTTACTCAG
	2	792	4.0	27,649	CGTTTACATCA
	3	186	3.5	5,388	CAGGTACAATG
	4	89	2.6	2,214	TGACTACACGG
	5	255	1.7	8,345	ACATTACTGCG

[a] Underlined letters represent the base with significant coverage increase
