# Supplementary material for: Characterization of MazF-Mediated Sequence-Specific RNA Cleavage in Pseudomonas putida Using Massive Parallel Sequencing
Source: PLoS One. 2016 Feb 17;11(2):e0149494. doi: 10.1371/journal.pone.0149494 (PMC4757574; doi:10.1371/journal.pone.0149494)
Supplement: S5 Table — (PDF) [file pone.0149494.s008.pdf]

Table S5

Number of occurrences	Number of the heptads
0	12188
1	3505
2	616
3	67
4	8
