# Supplementary material for: Characterization of MazF-Mediated Sequence-Specific RNA Cleavage in Pseudomonas putida Using Massive Parallel Sequencing
Source: PLoS One. 2016 Feb 17;11(2):e0149494. doi: 10.1371/journal.pone.0149494 (PMC4757574; doi:10.1371/journal.pone.0149494)
Supplement: S6 Table — (PDF) [file pone.0149494.s009.pdf]

Table S6

Number of occurrences	Number of the pentads
0	46
1	41
2	115
3	128
4	156
5	136
6	138
7	108
8	58
9	51
10	21
11	15
12	7
13	2
14	1
15	1
